# Age-related alterations in the oscillatory dynamics serving verbal working memory processing

**DOI:** 10.18632/aging.205403

**Published:** 2023-12-27

**Authors:** Seth D. Springer, Hannah J. Okelberry, Madelyn P. Willett, Hallie J. Johnson, Chloe E. Meehan, Mikki Schantell, Christine M. Embury, Maggie P. Rempe, Tony W. Wilson

**Affiliations:** 1 Institute for Human Neuroscience, Boys Town National Research Hospital, Boys Town, NE 68010, USA; 2 College of Medicine, University of Nebraska Medical Center, Omaha, NE 68198, USA; 3 Department of Neurology, Washington University School of Medicine, St. Louis, MO 63110, USA; 4 Department of Pharmacology and Neuroscience, Creighton University, Omaha, NE 68131, USA

**Keywords:** oscillation, magnetoencephalography, MEG, theta, alpha, aging

## Abstract

Working memory (WM) is a foundational cognitive function involving the temporary storage of information. Unfortunately, WM is also one of the most sensitive cognitive functions to the detrimental effects of aging. Expanding the field’s understanding of age-related WM changes is critical to advancing the development of strategies to mitigate age-related WM declines. In the current study, we investigated the neural mechanisms serving WM function in seventy-eight healthy aging adults (range: 20.2–65.2 years) using magnetoencephalography (MEG) and a Sternberg WM task with letter stimuli. Neural activity during the different phases of the WM task (i.e., encoding, maintenance, and retrieval) were imaged using a time-frequency resolved beamformer and whole-brain statistics were performed. We found stronger increases in theta activity and stronger decreases in alpha and beta activity (i.e., more negative relative to baseline) as a function of healthy aging. Specifically, age-related increases in theta activity were detected during the encoding period in the primary visual and left prefrontal cortices. Additionally, alpha and beta oscillations were stronger (i.e., more negative) during both encoding and maintenance in the left prefrontal cortex in older individuals. Finally, alpha and beta oscillations during the retrieval phase were stronger (i.e., more negative) in older participants within the prefrontal, parietal, and temporal cortices. Together, these results indicate that healthy aging strongly modulates the neural oscillatory dynamics serving WM function.

## INTRODUCTION

Working memory (WM) is a fundamental executive process which involves the storage, maintenance, and manipulation of relevant information. WM processes are generally grouped into three phases: information encoding, maintenance, and retrieval. The maintenance phase, which involves the rehearsal of the encoded information and the active inhibition of any new sensory stimuli, is the defining component which differentiates WM from other types of memory. Multiple studies have shown that WM processes are supported by a combination of neural spiking and oscillatory activity in the frontal and posterior cortices [1–8]. Historically, the neurophysiological underpinnings of WM function have been primarily investigated using functional MRI (fMRI). Such studies have revealed a left dominant, bilateral WM network with converging nodes in the dorsolateral prefrontal cortex (dlPFC), inferior frontal gyri, parieto-temporal cortices, and occipital regions [9–11]. More recently, electrophysiological studies have helped illuminate the specific oscillatory dynamics underlying WM function. Specifically, theta band activity during WM performance has been associated with the encoding of visual stimuli in primary visual cortices and higher-order cognitive control in the prefrontal cortex (PFC) [8, 12–14]. Similarly, alpha and beta band activity has been associated with higher-order visual processing in the posterior cortices and cognitive control in the PFC [14–17].

Previous literature has demonstrated that WM processes are vulnerable to age-related decline [18–20], with these changes being particularly evident at higher cognitive loads [21–23]. Across the adult lifespan, it has been shown that individuals tend to recruit the same brain regions when performing WM tasks [16, 24]. However, differential activation patterns become apparent when the cognitive load of the WM tasks is varied. For example, at lower cognitive loads, older individuals maintain WM performance comparable to younger individuals by recruiting more neural resources (i.e., stronger activations and/or more sites of activity) [24–26]. Nevertheless, at higher cognitive loads, this neural compensation in older individuals faulters, resulting in behavioral decrements accompanied by weakened neural activity [21, 24, 27–29]. This pattern of age-related overactivation, plateau, and subsequent underactivation is known as the compensation-related utilization of neural circuits hypothesis (CRUNCH) [30]. The CRUNCH phenomenon has been most commonly observed in PFC regions [21, 24, 27, 31–33], which are thought to be responsible for exerting executive control over WM processes [10, 11]. Further, these executive prefrontal areas have shown age-related differentiation in the lateralization of neural activity. Specifically, younger individuals consistently show stronger left hemispheric neural activity during WM performance, compared to a more bilateral pattern of activity in older adults [16, 24, 29, 31, 34, 35].

In addition to the plethora of past fMRI and positron-emission tomography (PET) work investigating age-related WM changes, more recent electrophysiologic work has been conducted to quantify the neural dynamics of these WM changes with age. One such study by Proskovec and colleagues [16] found that older adults had stronger alpha/beta oscillations (i.e., decreases in power relative to baseline), in right frontal regions during WM encoding and maintenance, and in the right superior temporal gyrus later in the maintenance phase. Conversely, it was shown that younger adults had stronger alpha/beta oscillations in the right parieto-temporal regions early in the encoding phase [16]. Age-related changes in alpha activity during WM processing was later shown by Tran and associates [36], who demonstrated that older adults had lower pre-trial alpha phase consistency, and that this lower phase consistency predicted lower WM task accuracy in older participants. Finally, event-related potential (ERP) analyses have revealed that, overall, older adults have reduced amplitudes in the fronto-central positivity (i.e., P200) and parietal positivity (i.e., P300) [37]. However, when high- and low-performing older adults were separately analyzed, high-performing adults had stronger P200 and P300 amplitudes, more similar to younger adults [38]. This interesting pattern of ERP results are in agreement with CRUNCH, with the high-performing older adults seemingly showing compensatory hyperactivation and the low-performing adults having reached a “resource ceiling” and decompensated.

Despite previous work characterizing age-related changes in WM, no previous studies have examined spectrally-specific age-related changes in neural oscillatory activity throughout all phases of WM (i.e., encoding, maintenance, and retrieval). Thus, in the current study we used the spatiotemporal precision of MEG to examine the effects of healthy aging on the spectrally-resolved neural oscillatory dynamics underlying all three phases of WM processing. We hypothesized that older adults would require stronger engagement of key left hemispheric frontal and parieto-occipital WM hubs. Additionally, we expected that prefrontal activity lateralization (i.e., stronger left hemispheric activity) during WM performance would diminish as a function of age, with older individuals tending to utilize a more bilaterally distributed WM network.

## METHODS

### Participants

Seventy-eight adults with a mean age of 45.10 (SD = 12.76) years were selected for inclusion in this study. The age range for males was 20.2 to 65.2 years and that for females was 21.4 to 62.2 years. These participants were chosen from a larger-scale study of accelerated aging in persons with HIV [39, 40], with only the HIV-negative participants included in this investigation of healthy aging. Of the 78 adults, 95% were right-handed, 79% were male, 10% were African-American, 6% were Asian, 77% were Caucasian, 4% were more than one race, and the remaining 3% preferred not to answer. This distribution corresponds closely to the racial demographics of the surrounding region. Notably, there was no effect of age on years of education (*r*_74_ = −.073, *p* = .531). Exclusionary criteria included any medical illness affecting CNS function (e.g., HIV/AIDS, Lupus, etc.), any neurological or psychiatric disorder, cognitive impairment, history of head trauma, current substance abuse, and the MEG laboratory’s standard exclusionary criteria (e.g., ferromagnetic implants). The Institutional Review Board reviewed and approved this investigation. Each participant provided written informed consent following a detailed description of the study.

### Experimental paradigm

**Figure 1:**
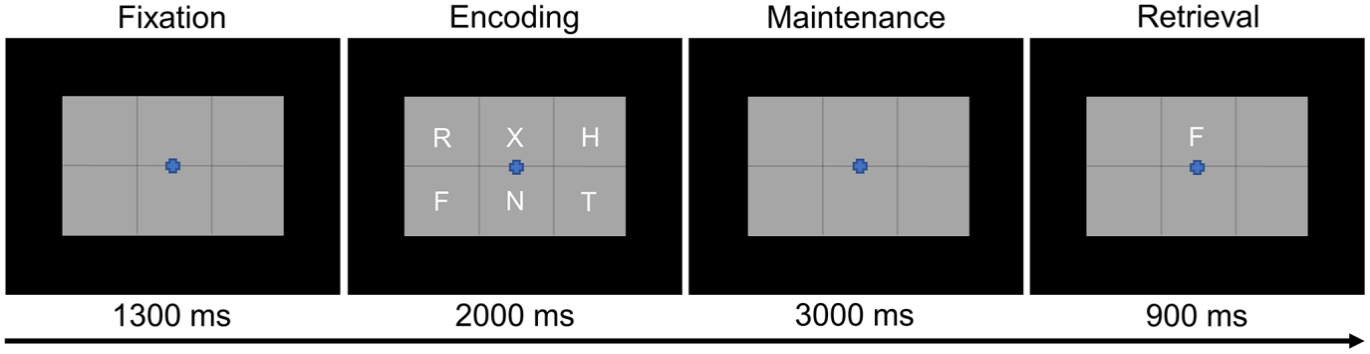
**Modified Sternberg working memory paradigm.** Participants were required to encode and maintain six letter stimuli. During the retrieval stage, participants were then required to indicate whether the probe letter was or was not present in the initial six letter set.

Participants were shown a centrally-presented fixation cross embedded in a 3 × 2 grid for 1.3 s (Figure 1). An array of six consonants then appeared at fixed locations within the grid for 2.0 s (i.e., encoding phase). Following the encoding phase, the letters disappeared and the empty grid remained on the screen for 3.0 s (i.e., maintenance phase). Finally, during the retrieval phase, a single probe consonant then appeared in the grid for 0.9 s, and the participant was instructed to respond via button press with their right index or middle finger as to whether the probe was in or out of the previous array of letters. A total of 128 trials were completed, equally split and pseudorandomized between in- and out-of-set trials, for a total run time of about 15 min. This task has been utilized in several previous studies from our laboratory [16, 17, 41–44].

### MEG data acquisition

All recordings were conducted in a magnetically-shielded room with active shielding engaged. Neuromagnetic responses were sampled continuously at 1 kHz, with an acquisition bandwidth of 0.1-330 Hz, using a MEGIN Vectorview MEG system with 306 magnetic sensors (MEGIN, Helsinki, Finland). During data acquisition, participants were monitored via real-time audio-visual feeds from inside the shielded room. Subject-wise MEG data were corrected for head motion and subjected to external noise reduction using signal space separation with a temporal extension [45].

### Structural MRI processing and MEG coregistration

Preceding MEG measurement, four head position indicator (HPI) coils were attached to the participant’s head and localized, together with three fiducial points and at least 100 scalp surface points, with a 3D digitizer (Fastrak 3SF0002, Polhemus Navigator Sciences, Colchester, VT, USA). Once in the MEG, electrical currents with unique frequencies (e.g., 322 Hz) were fed into each of the HPI coils. These HPI coil currents induced measurable magnetic fields, allowing the position of the HPI coils to be activity tracked relative to the MEG sensors throughout the recording. Since HPI coil locations are known in head coordinates, all MEG measurements could be transformed into a common coordinate system. With this coordinate system, MEG data were coregistered with each individual’s high-resolution structural T1-weighted MRI data prior to source reconstruction using Brain Electrical Source Analysis MRI (BESA MRI, Version 2.0, BESA GmbH, Gräfelfing, Germany). Individual structural MRI data for each participant was acquired using a Siemens Prisma 3T scanner (Siemens Medical Solutions) with a 64-channel head coil. An MP-RAGE sequence was utilized with the following parameters: TR: 2300 ms; TE = 2.98 ms; flip angle = 9°; FOV = 256 mm; slice thickness = 1.00 mm; voxel size = 1 × 1 × 1 mm. Structural MRI data were transformed into standardized space (i.e., Talairach space) and aligned parallel to the anterior and posterior commissures. Following source analysis, each participant’s MEG functional images were also transformed into standardized space and spatially resampled.

### MEG preprocessing, time-frequency transformation, and sensor-level statistics

Blink and cardiac artifacts were removed from the raw data using an adaptive artifact correction method in which brain activity is selectively separated from artifactual activities [46]. This adaptive artifact correction is accounted for during subsequent source analysis. The continuous magnetic time series was divided into 7200 ms epochs, with the baseline period being defined as the 400 ms prior to the encoding phase (i.e., −400 to 0 ms). Subsequently, epochs containing artifacts were removed based on a fixed threshold method, supplemented with visual inspection. Briefly, the amplitude and gradient distributions across all trials were determined per participant, and those trials containing the highest amplitude and/or gradient values relative to this distribution were rejected based on participant-specific thresholds. This approach was employed to minimize the impact of individual differences in sensor proximity to the brain and overall head size, which strongly affect MEG signal amplitude. Importantly, there were no age-related changes in either the amplitude (*r*_74_ = −.182, *p* = .115) or gradient (*r*_74_ = −.135, *p* = .245) thresholds used for artifact rejection. Artifact-free epochs were then transformed into the time-frequency domain using complex demodulation [47–49], with a resolution of 1 Hz and 50 ms between 2 and 100 Hz. Following time-frequency transformation, spectral power estimates per sensor were averaged across trials to generate plots of mean spectral density per sensor. These sensor-level data were then normalized to the baseline power within each frequency bin, which was calculated as the mean power for that 1 Hz bin during the −400 to 0 ms time period.

The significant time-frequency windows used for source imaging were determined by statistical analysis of the sensor-level spectrograms across the entire array of gradiometers. Briefly, each pixel per spectrograms was initially evaluated using a mass univariate approach based on the general linear model, followed by cluster-based permutation testing to address the problem of multiple comparisons [50, 51]. Specifically, a two-stage procedure was utilized to minimize false positive results while maintaining sensitivity. The first stage consisted of performing paired-sample *t*-tests against baseline on each pixel per spectrogram and thresholding the output spectrograms of *t*-values at *p* < .05 to define time-frequency bins containing potentially significant oscillatory deviations from baseline. Bins that survived thresholding (at *p* < .05) were clustered with temporally and/or spectrally neighboring bins that also survived, and cluster values were derived by summing all t-values within each cluster. In stage two, nonparametric permutation testing was used to derive a distribution of cluster-values and the significance level of the cluster(s) from stage one was tested directly using this permuted distribution, which was the result of 10,000 permutations. Based on this cluster-based permutation analysis, only the time-frequency windows that contained significant oscillatory deviations from baseline at the *p* < .001, corrected, threshold across all participants were subjected to source imaging (i.e., beamforming).

### MEG source imaging and statistics

Cortical networks were imaged through a time-frequency-resolved extension of the linearly constrained minimum variance (LCMV) beamformer [52–54]. The images were derived from the cross spectral densities of all combinations of MEG gradiometers averaged over the time-frequency range of interest, and the solution of the forward problem for each location on a grid specified by input voxel space. In principle, the beamformer operator generates a spatial filter for each grid point that passes signals without attenuation from a given neural region, while suppressing activity in all other brain areas. The filter properties arise from the forward solution (i.e., lead field matrix) for each location on a volumetric grid specified by input voxel space, and from the MEG cross spectral density matrix. Basically, for each voxel, a set of beamformer weights is determined, which amounts to each MEG sensor being allocated a sensitivity weighting for activity in the particular voxel. Following convention, the source power in these images was normalized per participant using a pre-stimulus period (i.e., baseline) of equal duration and bandwidth [55]. Such images are typically referred to as pseudo-t maps, with units (pseudo-t) that reflect noise-normalized power differences (i.e., active vs. passive) per voxel. MEG pre-processing and imaging used BESA (version 7.0) software.

After imaging, average whole-brain maps were computed across all participants for the selected time-frequency windows. These 3D maps of brain activity were used to assess the neuroanatomical basis of the significant oscillatory responses identified through the sensor-level analysis. Finally, these source images were subjected to correlation analyses with age to investigate how the oscillatory processes underlying WM encoding, maintenance, and retrieval change as a function of age. To control the multiple comparisons inherent to whole-brain voxel-wise statistics, cluster-based permutation testing was again utilized [50, 51, 56]. This whole-brain cluster-based permutation testing was virtually identical to the approach used for sensor-level statistics (see above), with the exception that the input data was voxel-level neural responses and we used a different statistical threshold following permutation testing (i.e., stage one threshold of *p* < .001; threshold of *p* < .05 following permutation testing for multiple comparisons correction).

### Statistical analyses

Statistical analyses were performed using custom R [57] and MATLAB [58] scripts. Correlation analyses were used to test differences as a function of healthy aging in education, accuracy, reaction time, total correct trials, amplitude cutoffs, and gradient cutoffs. Permutation testing of the sensor-level spectrograms and whole-brain images was performed using BESA Statistics (Version 2.1, BESA GmbH, Gräfelfing, Germany). Finally, to reduce the impact of outliers on statistical analyses, participants with values 2.5 SDs above or below the group mean were excluded for each analysis.

### Data availability

The data that support the findings of this study are available from the corresponding author, upon reasonable request. Specifically, the anonymized data will be available for non-commercial research purposes and responses will occur at earliest convenience with a goal of delivering the data within 1 month.

## RESULTS

### Behavioral results

Of the 78 participants that performed the verbal WM task, two were removed due to poor task performance (i.e., <65% correct). While there was no effect of age on accuracy (*r*_74_ = .012, *p* = .919), there was an effect of age on reaction time (*r*_74_ = .453, *p* < .001) such that older participants were slower than younger participants. Across all participants, the mean accuracy was 84.33% (SD = 7.04%) and the mean reaction time was 895.4 ms (SD = 193.06 ms).

### Spectrally-specific neural oscillations

**Figure 2:**
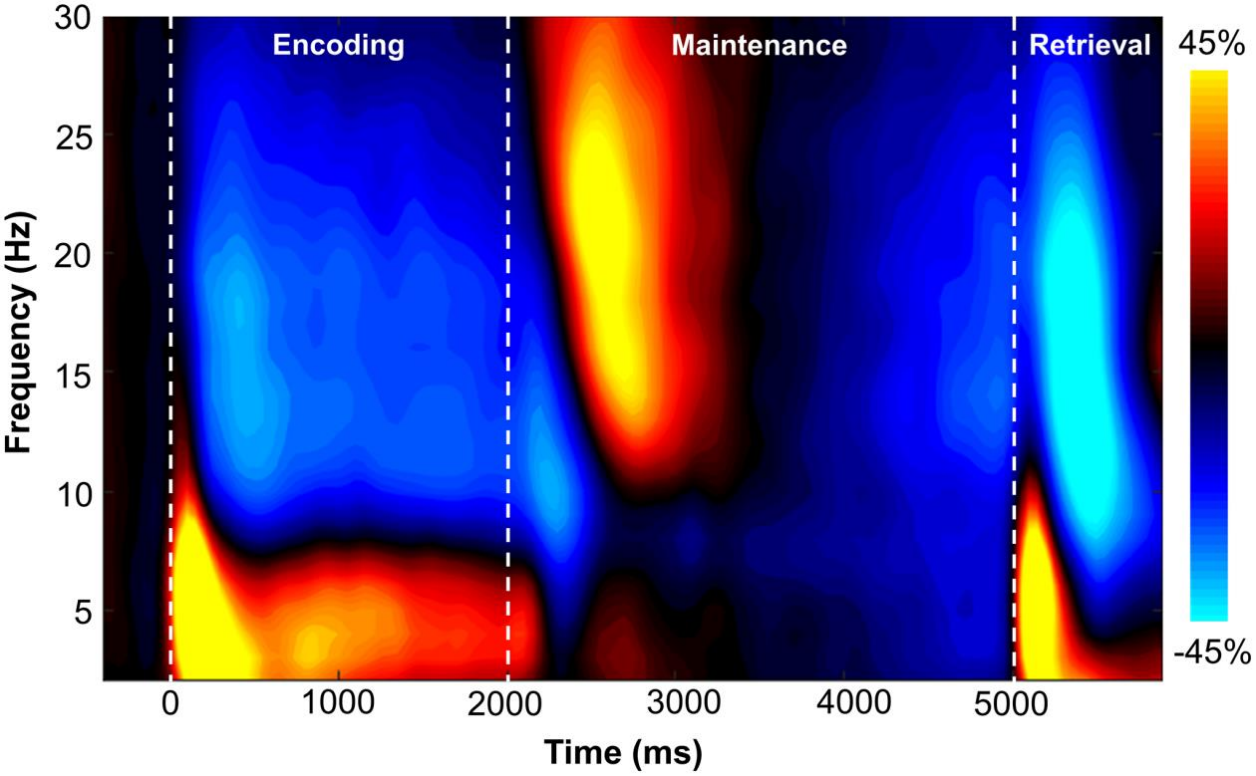
**Grand-averaged time-frequency spectrogram from a sensor near parieto-occipital areas (MEG1922) with time (ms) shown on the x-axis and frequency (Hz) denoted on the y-axis.** A color scale bar is shown to the right of the spectrogram which shows the percent power change relative to the baseline period (−400 to 0 ms).

Sensor-level time-frequency analysis across all participants revealed significant clusters (*p* < .001, corrected) of theta, alpha, and beta band oscillatory activity during the encoding, maintenance, and retrieval phases (Figure 2). During the encoding phase, theta band (3-6 Hz) activity sharply increased immediately following stimulus presentation (i.e., 0 ms), continued through the encoding phase, and slowly dissipated over the first 800 ms of the maintenance phase (i.e., 3-6 Hz, 0-2800 ms; *p* < .001, corrected). Additionally, decreases in alpha band (9-15 Hz) activity began around 200 ms after the onset of the encoding grid and were sustained throughout the encoding phase, terminating near the beginning of the maintenance phase (i.e., 9-15 Hz, 200-1800 ms; *p* < .001, corrected). During the maintenance phase, there was a significant cluster of increased alpha/beta activity in a slightly higher frequency band (12-17 Hz; 2500-2900 ms; *p* < .001, corrected). During the retrieval phase, an increase in theta activity followed retrieval probe presentation (3-6 Hz; 5000-5350 ms; *p* < .001, corrected). Finally, overlapping decreases in alpha (8-12 Hz; 5350-5750 ms) and beta (14-19 Hz; 5250-5650 ms) band activity were observed during the retrieval phase (both ps < .001, corrected).

### Beamformer analysis

**Figure 3:**
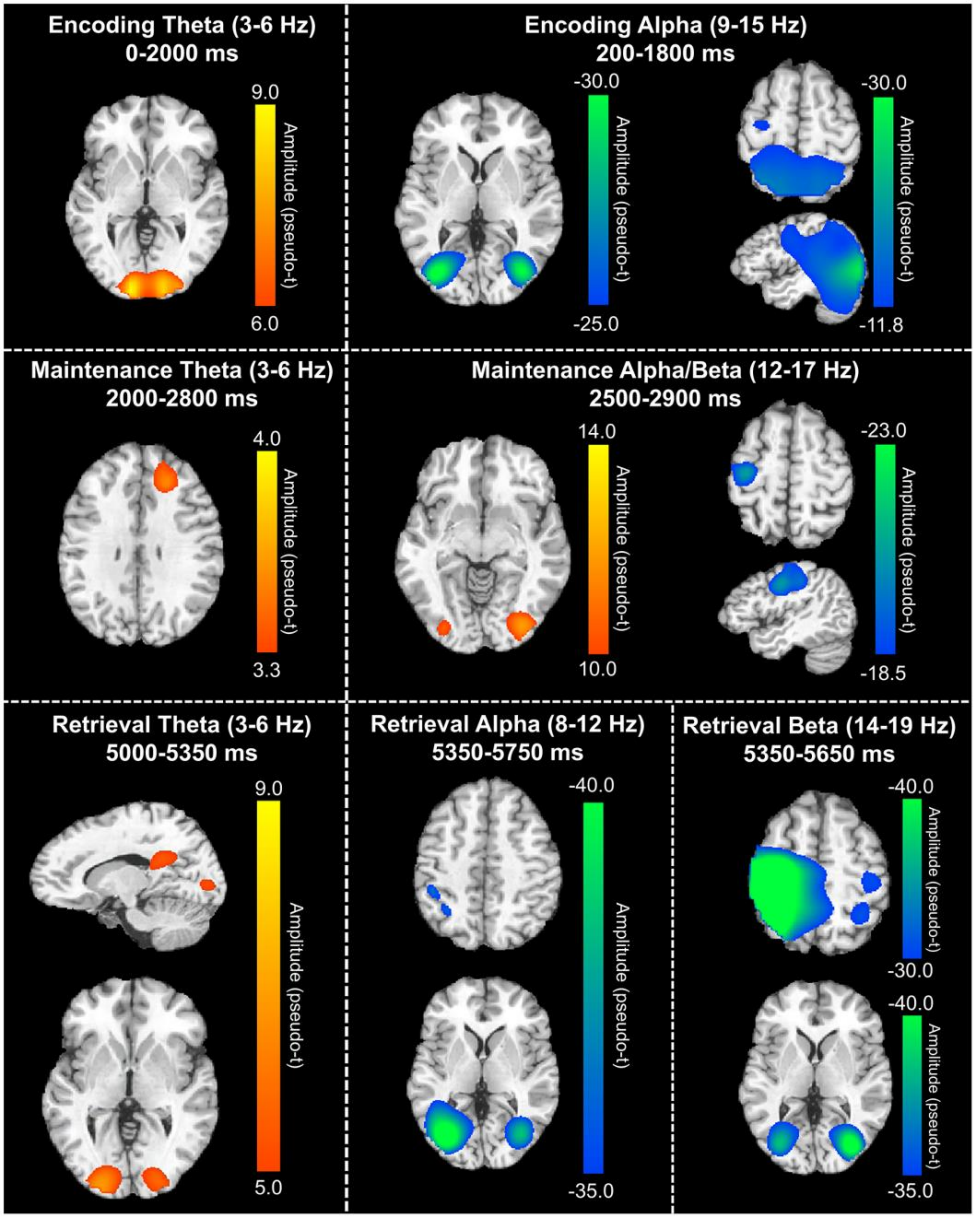
**Grand-averaged beamformer images (pseudo-t) across all participants for theta (left) and alpha/beta (right) bands.** During the encoding phase (top), there were increases in theta band amplitude relative to baseline in the primary visual cortex and decreases in alpha band activity (i.e., more negative relative to baseline) in the lateral occipital cortices, parietal cortices, and left hemisphere frontal cortex (including language areas). During the maintenance phase (middle), theta increases were limited to the right dlPFC and decreased alpha/beta activity in the left hemisphere frontal cortex (relative to baseline) was largely sustained. Additionally, a decrease in alpha/band activity relative to the baseline was observed in the lateral occipital cortices during the maintenance phase. During the retrieval phase (bottom), theta band activity increased in the right posterior cingulate gyrus and bilateral primary visual cortices, while alpha activity decreased (i.e., became more negative) in the lateral occipital cortices, left SMG, and left angular gyrus. Finally, decreased beta activity (i.e., more negative relative to baseline) in the bilateral primary motor cortices, lateral occipital cortices, and right superior parietal lobe was observed.

In order to determine the neural regions involved in verbal WM, the aforementioned sensor-level time-frequency bins of interest for theta, alpha, and beta band oscillatory activity were imaged using a time-frequency-resolved beamformer. Considering that beamforming windows must be the same length as the baseline period (i.e., 400 ms), the oscillatory deviations from baseline were imaged in 400 ms increments or less. For the temporally extended oscillatory responses (i.e., those over 400 ms), participant-level image averaging was performed for each neural response as follows: theta encoding (3-6 Hz; 0-2000 ms), theta maintenance (3-6 Hz; 2000-2800 ms), and alpha encoding (9-15 Hz; 200-1800 ms). These whole-brain average maps per participant were then averaged across all individuals to allow for visualization of the neural oscillations during the different WM phases (i.e., encoding, maintenance, and retrieval). These images revealed differential patterns of neural activity in the theta, alpha, and beta bands throughout all phases of WM performance (Figure 3). During the encoding phase, a strong increase in theta activity relative to the baseline was observed in the bilateral primary visual cortices, while decreased alpha activity (i.e., more negative relative to baseline) was observed in the lateral occipital cortices bilaterally, and left frontal cortex. During the maintenance phase, increased theta activity was observed in the right dorsolateral prefrontal cortex (dlPFC). Further, the decreased alpha/beta oscillations (i.e., more negative) observed in the frontal cortices during the encoding phase were sustained through the maintenance phase, along with decreased alpha activity in the lateral occipital cortices bilaterally. During the retrieval phase, theta band activity was shown to strongly increase in the right posterior cingulate gyrus and the bilateral primary visual cortices. Decreased alpha activity (i.e., more negative) during the retrieval phase were observed in the lateral occipital cortices, left supramarginal gyrus (SMG), and left angular gyrus. Similarly, decreased beta oscillations (i.e., more negative) during the retrieval phase were found in the lateral occipital cortices, bilateral primary motor cortices, and bilateral superior parietal cortices.

**Figure 4:**
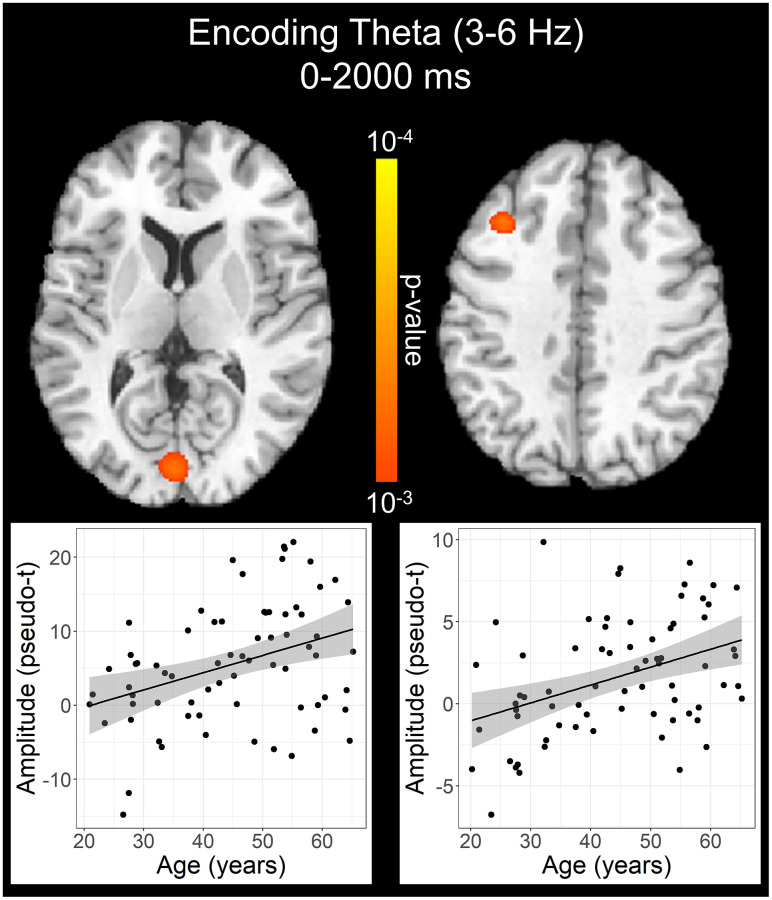
**Effect of age on theta oscillations during the encoding phase.** Whole-brain linear regression analysis revealed that theta activity increased in the left primary visual cortex and left dlPFC with older age. Linear regression plots of peak voxel pseudo-t values are shown as a function of age. Lines of best-fit and 95% CI (shaded area) are overlaid.

**Figure 5:**
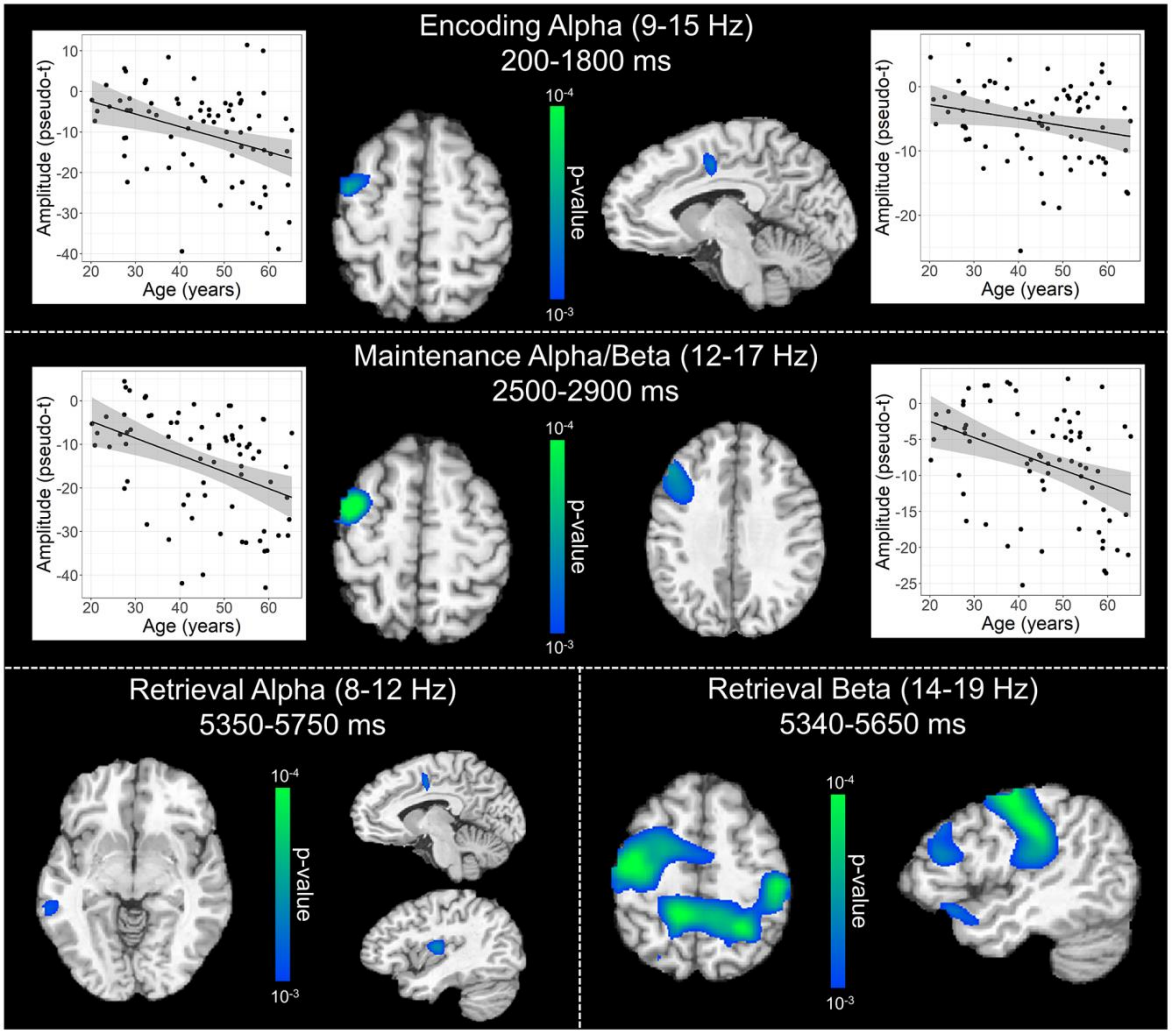
**Effect of age on alpha and beta oscillations during the encoding, maintenance, and retrieval phases.** Whole-brain linear regression analyses testing for age-related changes in alpha (encoding and retrieval), alpha/beta (maintenance), and beta (retrieval) are shown during the encoding (top), maintenance (middle), and retrieval bottom) phases. During encoding (top), decreases in alpha activity became significantly stronger (i.e., more negative relative to baseline), as a function of age, in prefrontal cortical regions and anterior cingulate. Additionally, age-related alterations in alpha and beta activity during the maintenance period (middle) were found in the left prefrontal cortices, while age-related effects during retrieval (bottom) were observed across a diverse set of brain regions that have been implicated in working memory processing (i.e., frontal, temporal, and parietal cortices). Beyond statistical maps, linear regression plots of peak voxel pseudo-t values are shown as a function of age for encoding and maintenance peaks. Lines of best-fit and 95% CI (shaded area) are overlaid.

To statistically examine the effects of healthy aging on the neural dynamics serving WM function, we subjected the frequency-specific whole-brain encoding, maintenance, and retrieval participant-level average maps to correlation analysis. These whole-brain correlation analyses were then followed by cluster-based permutation testing to correct for multiple comparisons. These analyses revealed that theta oscillations during encoding became significantly stronger as a function of age in the left primary visual cortex (*p* = .037, corrected) and left dlPFC (*p* = .045, corrected; Figure 4), and that there were no age-related theta effects during the maintenance or retrieval phases. In contrast, the decreases in alpha and beta became stronger (i.e., more negative) with increasing age throughout all phases of WM processing (i.e., encoding, maintenance, and retrieval; Figure 5). During the encoding phase, decreases in alpha became stronger (i.e., more negative) as a function of age in the left middle frontal gyrus (*p* = .023, corrected) and the left anterior cingulate gyrus (*p* = .033, corrected). This age-related change in activity in the left middle frontal gyrus was sustained through the maintenance phase (*p* = .029, corrected), along with stronger decreases in alpha/band responses (i.e., more negative) in the left dlPFC (*p* = .031, corrected). During the retrieval phase, decreases in alpha oscillations also became stronger (i.e., more negative) as a function of age in the left insula (*p* = .009, corrected), left anterior cingulate (*p* = .020, corrected), and left middle temporal gyrus (*p* = .031, corrected). Finally, the decreases in beta oscillations (14-19 Hz) during the retrieval phase were found to get stronger (i.e., more negative) with increasing age in the left primary motor cortex (*p* < .001, corrected), right primary somatosensory cortex (*p* < .001, corrected), bilateral superior parietal cortices (ps < .001, corrected), left dlPFC (*p* = .007, corrected), and left anterior superior temporal gyrus (*p* = .020, corrected). Alpha and beta retrieval phase scatterplots that were not included in Figure 5 can be found in the Supplementary Material (Supplementary Figure 1). A complete summary of all significant effects can be found in Table 1.

**Table 1:** Coordinates of the peak response in each significant age correlation cluster.

**Region/Effect of interest**	**Frequency**	**X**	**Y**	**Z**	***df***	**Statistic**
***Encoding***						***r***
Left primary visual cortex	θ	−2	−85	10	70	.42
Left dlPFC	θ	−34	24	38	70	.40
Left middle frontal gyrus	α	−42	−5	54	74	−.41
Left anterior cingulate gyrus	α	−10	−5	42	74	−.40
***Maintenance***						***r***
Left middle frontal gyrus	α/β	−42	−1	54	72	−.42
Left dlPFC	α/β	−42	28	42	72	−.42
***Retrieval***						***r***
Left insula	α	−30	−13	10	72	−.41
Left anterior cingulate gyrus	α	−6	−5	42	72	−.40
Left middle temporal gyrus	α	−58	−37	-3	72	−.39
Left primary motor cortex	β	−42	−13	50	71	−.45
Right primary somatosensory cortex	β	42	−21	42	71	−.46
Left superior parietal cortex	β	−14	−45	54	71	−.45
Right superior parietal cortex	β	30	−53	58	71	−.45
Left dlPFC	β	−50	32	22	71	−.41
Left anterior superior temporal gyrus	β	−50	24	-11	71	−.41

All test statistics are significant at *p* < .001 level and survive cluster-based permutation testing. All coordinates are in Talairach space. Abbreviations: dlPFC: dorsolateral prefrontal cortex; *df*: degrees of freedom.

## DISCUSSION

In the present study, we investigated the effect of healthy aging on the neural oscillatory dynamics serving verbal WM processing using MEG and advanced source reconstruction. Similar to previous studies utilizing a modified Sternberg WM task in healthy aging adults, we found that reaction time increased with age and that there were no significant aging effects on task accuracy [16, 59]. Across all participants, our results also showed that WM processing is supported by regionally distinct neural oscillations in the theta, alpha, and beta bands throughout all phases (i.e., encoding, maintenance, and retrieval) and that such activity is broadly affected by healthy aging. Briefly, theta oscillations were shown to become stronger in the primary visual cortices and in the frontal cortex with increasing age. Furthermore, throughout all WM phases and in several frontal, parietal, and temporal regions, alpha, beta, and alpha/beta activity became stronger (i.e., more negative) as a function of age. Critically, these results represent the first oscillatory analysis of verbal WM function in a healthy aging sample in which neural activity was examined throughout all phases of processing, including the retrieval stage. Below, we discuss the implications of these novel findings on our understanding of how healthy aging affects verbal WM processing.

Across all frequencies, our findings demonstrate widespread and robust cortical activation patterns during verbal working memory task performance, as well as age-related changes in these neural activations.

These task-related activations and age-related changes are most prevalent in the occipital, parietal, and frontal cortices; regions which have been shown to be critical to verbal working memory performance [9, 10]. According to the theories of working memory function originally proposed by Baddeley and colleagues, the neural subsystems serving working memory processing include the visuospatial sketchpad, phonological loop, episodic buffer, and the central executive [60, 61]. The visuospatial sketchpad is responsible for processing the visual and spatial components of a stimulus, and is served by the occipital and parieto-temporal cortices [9, 10]. The phonological loop deals with the processing and rehearsal of auditory and language information. Further, the phonological loop is a predominately left hemispheric network comprised of the phonological store in the parietal lobe [9, 62], which briefly stores fleeting memory traces, and the articulatory process in the inferior frontal gyrus and superior temporal gyrus (e.g., Broca’s area) [63–65], responsible for the rehearsal and manipulation of information in the phonological store (e.g., through subvocal repetition). The episodic buffer is a more distributed subsystem which links working memory and long-term memory, facilitating the integration of information from separate modalities (i.e., visual, spatial, auditory, language, etc.) and binding information into coherent episodes [61]. Finally, the central executive component exerts top-down control over the other working memory subsystems, via the allocation of attentional resources and direction of information flow to the other subsystems, and is thought be housed in the prefrontal cortex [10, 11]. Considering the letter-based stimuli utilized in our modified Sternberg working memory task, we expected robust activation of each of the aforementioned subsystems.

One of our most interesting findings was the broad strengthening of alpha and beta oscillations (i.e., decreases from baseline) with increasing age. The local suppression of alpha and beta activity, relative to baseline levels, has been shown to represent active cortical engagement (i.e., active visual processing in higher-order visual areas) [16, 17, 66–68]. This is further supported by multimodal studies which have demonstrated co-localization of decreases in alpha and beta activity with increased BOLD fMRI signal during cognitive tasks [69, 70]. During the encoding phase, older individuals showed significantly stronger decreases in alpha activity (i.e., more negative relative to baseline) in the left middle frontal gyrus (MFG) and the left anterior cingulate. This significant age-related shift in left MFG activity extended into the maintenance period, with the addition of age-related effects in alpha/beta activity in the left dlPFC. These prefrontal cortical regions are thought to act as central executive nodes during WM task performance, allowing for the allocation of WM subsystem resources and the direction of attention [10, 11]. These findings of age-related increases in frontal regions are in widespread agreement with the aging literature, which has demonstrated that increased prefrontal neural recruitment can offset declines in cognitive performance as a function of healthy aging (i.e., CRUNCH) [21, 24, 27, 30–33]. Of note, we found no significant age-related changes during the encoding and maintenance phases in alpha/beta activity in either visual processing or language areas, which have historically been linked to the visuospatial sketchpad and phonological loop, respectively [9, 10, 62]. In sum, the present work is in agreement with our hypotheses that older adults would require stronger engagement of left hemispheric frontal WM hubs and reinforces previous aging WM literature in demonstrating how crucial increased neural activity in prefrontal cortices is for older individuals during WM processing.

Beyond the encoding and maintenance phases, we found significant alpha and beta oscillatory changes as a function of age during the retrieval phase. Though previous studies have extensively characterized neural activity during WM encoding and maintenance, studies analyzing the neural dynamics during the retrieval phase are sparse. During the retrieval phase, older participants showed stronger decreases in alpha activity (i.e., more negative) in the left posterior middle temporal gyrus (MTG), left insula, and left anterior cingulate. Additionally, beta activity during the retrieval phase decreased (i.e., more negative) with increasing age in the left primary motor cortex, bilateral superior parietal cortices, left dlPFC, left anterior superior temporal gyrus, and other regions. Thus, unlike the age-related alpha/beta effects observed during the encoding and maintenance phases, which were largely confined to prefrontal cortices, age-related changes in alpha/beta activity during the retrieval phase were found to extend to other WM subsystems. Specifically, in the retrieval phase, older individuals tended to more strongly engage neural regions linked to higher-order visual processing (i.e., visuospatial sketchpad; left posterior MTG), language processing (i.e., left STG), phonological storage (i.e., superior parietal lobes), central executive processes (i.e., left dlPFC), and motor execution (i.e., primary motor cortex). These retrieval phase findings are in line with our hypothesis that older adults would more strongly recruit left hemispheric frontal and parieto-occipital WM nodes, relative to younger participants. As noted above, only a few studies have examined the retrieval stage, Guran and associates [71] found that older (i.e., 53-80 years) compared to younger (i.e., 18-30 years) individuals had stronger alpha/beta decreases relative to baseline in sensors overlying frontal, central, and parietal regions [71], which is consistent with our findings. Finally, our finding of stronger decreases in beta (i.e., more negative) in the primary motor cortex in older participants is consistent with work from the motor control literature [72, 73]. Thus, this effect of aging on motor beta activity is likely task independent.

We also found robust age-related effects in the theta range. Theta oscillations in the primary visual cortex have been widely implicated in basic visual processing, while frontal theta activity has been shown to be important for the higher-order organization and maintenance of stimulus information [8, 12–14, 66, 74]. Though theta activity has been shown to be critical for WM function, age-related changes in these theta band dynamics have only been weakly characterized. Age-related theta alterations in the current study were found to be confined to the encoding phase, with activity in the primary visual cortex and left dlPFC getting stronger as a function of increasing age. One comparable study found that older participants (i.e., 62–71 years) were more susceptible to WM disruption and that frontal midline theta was stronger in older individuals (i.e., 19–29 years) [75]. Our findings of age-related increases in theta activity, particularly those in the dlPFC, are consistent with our alpha and beta band findings and the neural compensation hypothesis (i.e., CRUNCH). Additionally, theta band activity in the PFC of healthy young adults during WM performance has been shown to increase stepwise with increasing task load [12, 14, 66], further supporting the idea that the increased theta activity with increasing age may represent these older individuals needing to exert more neural resources to complete this WM task (i.e., less efficient processing). Altogether, in conjunction with our alpha and beta findings, we have demonstrated that the widely cited age-related increases in neural recruitment are not frequency specific, with age-related effects spanning multiple frequency bands (i.e., theta, alpha, and beta) and working memory subsystems.

Interestingly, our results did not show the hypothesized and commonly reported age-related change in the lateralization of prefrontal activation. Specifically, previous work has demonstrated that younger individuals more strongly utilize left hemispheric prefrontal regions during working memory performance, compared to older individuals who exhibit more bilateral prefrontal activity [16, 24, 29, 31, 34, 35]. This discrepancy may be due to study design and analysis differences. Firstly, our sample of 78 health adults is nearly double the size of most previous healthy aging WM studies and our whole-brain data were modeled using age as a continuous variable, compared to dichotomizing age groups as has commonly been done [16, 24, 31, 35]. Secondly, the current study utilized cluster-based permutation testing of the source images to strongly control for false positives, compared to the much less stringent multiple comparison correction approaches that have previously been used (e.g., uncorrected or cluster-size corrections) [16, 31, 35]. Finally, the oldest participants in the current study were younger (i.e., 65 years) than many aging WM studies (e.g., 75–82 years) [24, 31, 34, 35]. Thus, our relatively younger participants may not have reached a level of cognitive dysfunction that necessitates bilateral prefrontal recruitment, but future studies are needed to confirm these findings.

Before closing, it is important to note the limitations of this study. First, as mentioned above, the oldest participants in our sample were slightly younger (i.e., 65 years) than some previous WM studies of aging (e.g., 75–82 years). For future studies, increasing the age of the oldest participants would allow for the hypothesized transition from age-related compensation (i.e., increased neural activity and normal accuracy) to age-related decompensation (i.e., decreased neural activity and decreased accuracy) to be more thoroughly studied. Finally, the modified Sternberg WM paradigm that was utilized for the current study required that participants remember six letter stimuli per trial and previous work has shown that varying the cognitive load of WM tasks leads to an interesting pattern of effects in older individuals (i.e., greater neural activity at low working memory loads with decompensation and decreased neural activity at higher working memory loads) [21, 24, 27–29]. Thus, future aging WM research would benefit from having multiple memory load conditions of varying difficulty.

Taken together, these data support the previous literature showing greater recruitment of the prefrontal cortex in older individuals during WM performance. Importantly, our findings extend this literature by demonstrating that age-related hyperactivation of prefrontal regions occurs in all phases of WM (i.e., encoding, maintenance, and retrieval) and is multispectral involving theta, alpha, and beta oscillatory activity. Further, though the age-related changes in alpha and beta band activity were restricted to prefrontal executive control regions during the encoding and maintenance phases, these age-related changes emerged in a more widespread network that included language (i.e., phonological loop) and motor regions during the retrieval phase. Developing a better understanding of these age-related alterations in WM function may have important implications for developing targeted interventions aimed at improving cognitive function and promoting healthy aging across the lifespan.

## Supplementary Materials

Supplementary Figure 1
