# Supplementary material for: Age-related alterations in the oscillatory dynamics serving verbal working memory processing
Source: Aging (Albany NY). 2023 Dec 27;15(24):14574–90. doi: 10.18632/aging.205403 (PMC10781444; doi:10.18632/aging.205403)
Supplement: Supplementary Figure 1 [file aging-15-205403-s001.pdf]

SUPPLEMENTARY FIGURE

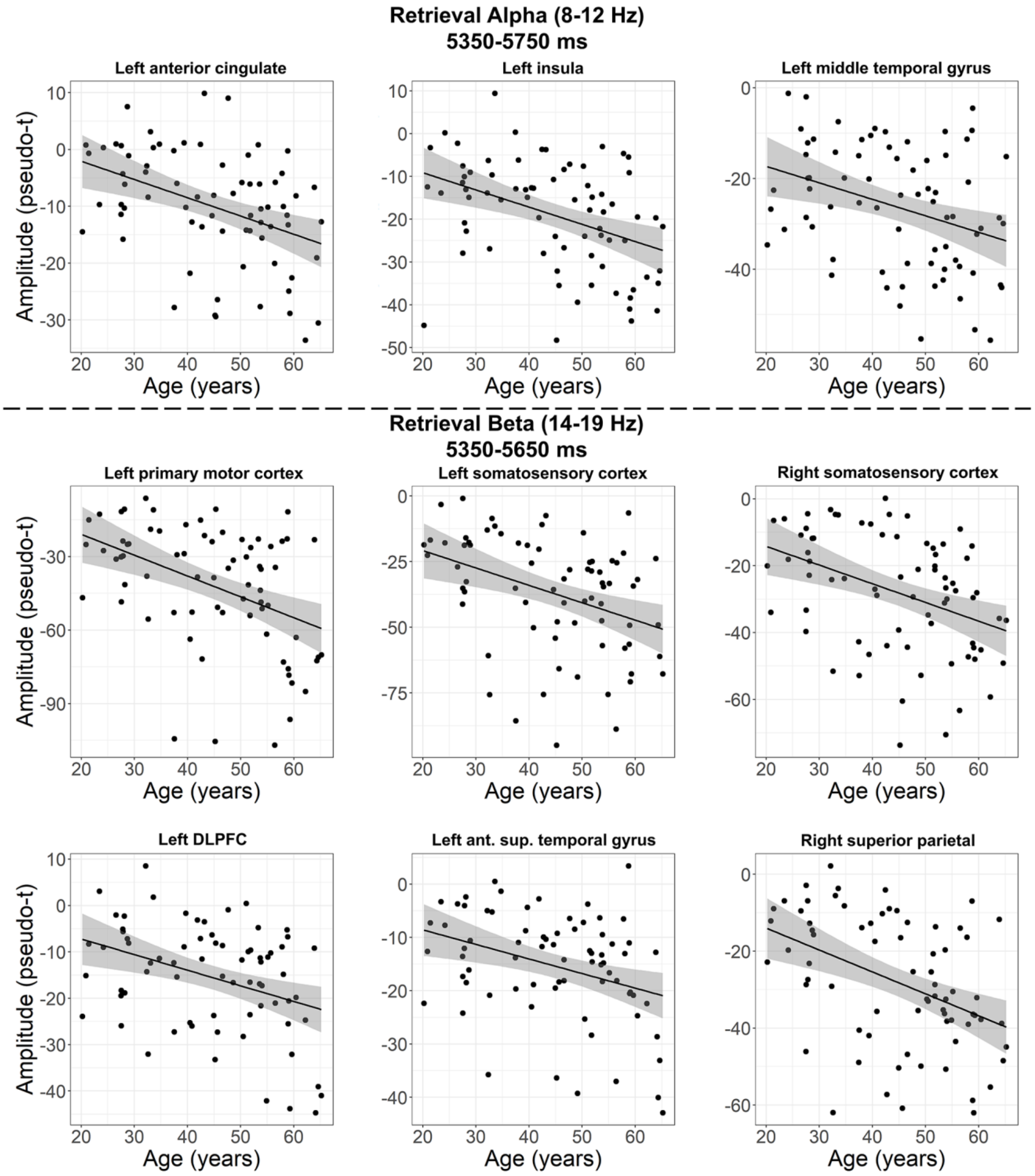

Supplementary Figure 1. Linear regression plots of peak voxel pseudo-t values are shown as a function of age for the alpha and beta retrieval peaks from Figure 5.
